# Phosphoinositide Conversion Inactivates R‐RAS and Drives Metastases in Breast Cancer

**DOI:** 10.1002/advs.202103249

**Published:** 2022-01-31

**Authors:** Huayi Li, Lorenzo Prever, Myriam Y. Hsu, Wen‐Ting Lo, Jean Piero Margaria, Maria Chiara De Santis, Cristina Zanini, Marco Forni, Francesco Novelli, Salvatore Pece, Pier Paolo Di Fiore, Paolo Ettore Porporato, Miriam Martini, Hassane Belabed, Marc Nazare, Volker Haucke, Federico Gulluni, Emilio Hirsch

**Affiliations:** ^1^ Department of Molecular Biotechnology and Health Sciences University of Turin Turin 10126 Italy; ^2^ Leibniz‐Forschungsinstitut für Molekulare Pharmakologie (FMP) Berlin 13125 Germany; ^3^ IEO European Institute of Oncology IRCCS Via Ripamonti 435 Milan 20141 Italy; ^4^ Department of Oncology and Hemato‐Oncology Università degli Studi di Milano Milano 20142 Italy; ^5^ Faculty of Biology, Chemistry and Pharmacy Freie Universität Berlin Berlin 14195 Germany

**Keywords:** breast cancer, focal adhesions, metastases, migration, PI3KC2*α*, RASA3, R‐RAS

## Abstract

Breast cancer is the most prevalent cancer and a major cause of death in women worldwide. Although early diagnosis and therapeutic intervention significantly improve patient survival rate, metastasis still accounts for most deaths. Here it is reported that, in a cohort of more than 2000 patients with breast cancer, overexpression of PI3KC2*α* occurs in 52% of cases and correlates with high tumor grade as well as increased probability of distant metastatic events, irrespective of the subtype. Mechanistically, it is demonstrated that PI3KC2*α* synthetizes a pool of PI(3,4)P2 at focal adhesions that lowers their stability and directs breast cancer cell migration, invasion, and metastasis. PI(3,4)P2 locally produced by PI3KC2*α* at focal adhesions recruits the Ras GTPase activating protein 3 (RASA3), which inactivates R‐RAS, leading to increased focal adhesion turnover, migration, and invasion both in vitro and in vivo. Proof‐of‐concept is eventually provided that inhibiting PI3KC2*α* or lowering RASA3 activity at focal adhesions significantly reduces the metastatic burden in PI3KC2*α*‐overexpressing breast cancer, thereby suggesting a novel strategy for anti‐breast cancer therapy.

## Graphical abstract

The authors report that overexpression of PI3KC2α in breast cancer correlates with high tumor grade and increased probability of distant metastatic events. Mechanistically, PI3KC2α produces PI(3,4)P2 at focal adhesion which inactivates R‐RAS by recruiting RASA3. This leads to increased focal adhesion disassembly and cell migration. Proof‐of‐concept is provided that inhibiting PI3KC2α or lowering RASA3 significantly reduces the metastatic burden.

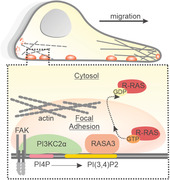

## 1 Introduction

Metastasis is a leading cause of mortality in cancer patients which results from a multistep process characterized by increased migration and invasion of cancer cells.^[^ ^1^ ^]^ Such phenotype requires the rearrangement of the cytoskeleton and abnormal cell adhesion including disassembly of focal adhesions at the rear of the cells.^[^ ^2^ ^]^ Focal adhesions (FAs) are large macromolecular complexes that link actin cytoskeleton to the extracellular matrix to provide traction, thereby enabling cell migration.^[^ ^3^ ^]^ In normal and malignant cells, focal adhesion kinase (FAK), a non‐receptor tyrosine kinase, is an essential regulator of focal adhesions through its kinase activity and scaffolding functions.^[^ ^4^ ^]^ Increased FAK expression and activity are often associated with metastasis and poor clinical outcome in cancer patients, highlighting FAK as a potential determinant of cancer progression.^[^ ^5^ ^]^ However, the molecular mechanisms controlling FAK activation and focal adhesion dynamics in breast cancer metastasis remain elusive.

Phosphoinositides (PIs) are lipid signaling molecules with fundamental roles in many aspects of cell physiology.^[^ ^6^ ^]^ PIs, together with GTPases, promote the anchoring of effectors to distinct membranes and mediate the activation of both GEFs (guanine nucleotide exchange factors) and GAPs (GTPase‐activating proteins) after membrane‐association through their PI‐binding sites.^[^ ^7^ ^]^ Increasing evidence suggests that phosphatidylinositol 3,4‐bisphosphate [PI(3,4)P2], can regulate membrane ruffle,^[^ ^8^ ^]^, podosome,^[^ ^9^ ^]^ lamellipodia, and invadopodia formation and maturation^[^ ^10^ ^]^ in different cellular models. For example, lack of PI(3,4)P2 alters focal adhesion dynamics in MDA‐MB‐231 breast cancer cells.^[^ ^11^ ^]^ Similarly, class II PI3K *α* (PI3KC2*α*), a lipid kinase synthetizing PI(3,4)P2 mostly at the plasma membrane, is overexpressed and associated with poor prognosis in breast cancer.^[^ ^12^ ^]^ Whether PI3KC2*α* overexpression and increased PI(3,4)P2 production in breast cancer increase metastatic spreading is unclear.

Here, we unveil a PI(3,4)P2‐selective role in the control of focal adhesion stability in breast cancer cells. Based on our recently solved PI3KC2*α* crystal structure, we identified a helical bundle domain (HBD) in the N‐terminal region of PI3KC2*α* that is directly involved in its localization to focal adhesions. In cells overexpressing PI3KC2*α*, PI(3,4)P2 accumulates at focal adhesions where it recruits the Ras‐GAP, RASA3 (GAP1IP4BP, R‐Ras GAP). Increased localization of RASA3 at focal adhesions leads to R‐RAS inactivation and reduced focal adhesion stability, promoting cell migration and invasion. Finally, our data indicate that reduction in RASA3 activity is sufficient to prevent metastasis in both cellular and animal models of breast cancer.

## 2 Results

### 2.1 PI3KC2*α* Overexpression in Breast Cancer Leads to Increased Cell Migration, Invasion and Metastasis

**Figure 1:**
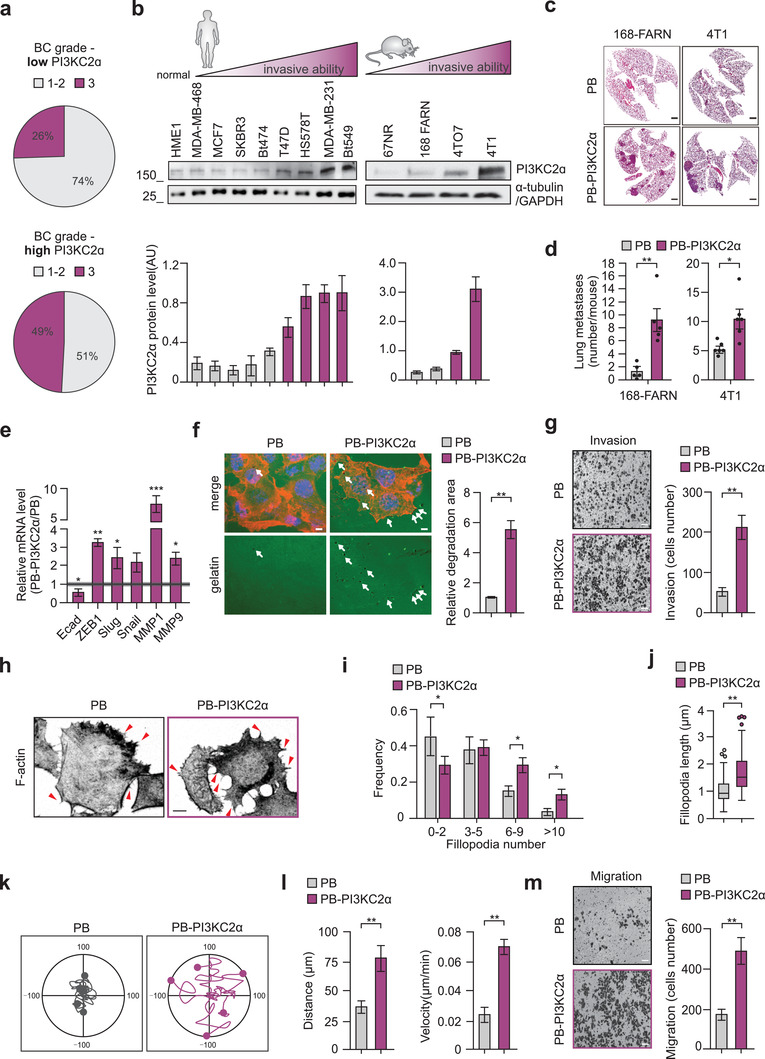
Overexpression of PI3KC2*α* in breast cancer leads to increased metastatic burden and cell migration. a) Pie chart showing breast cancer grade in either low or high PI3KC2*α* patients extracted from Gulluni et al. Table 1 ^[^ ^12^ ^]^. b) Quantification and representative blots showing PI3KC2*α* protein expression normalized over *α*‐tubulin in human BC cell lines and normal mammary cell line HME1 (left panels). Quantification and representative blots showing PI3KC2*α* protein expression normalized over GAPDH in four isogenic murine BC cell lines (right panels). c,d) Lung metastasis analyzed in BALB/c mice orthotopically injected with PB or PB‐PI3KC2*α* 168‐FARN (left panels). Lung metastasis analyzed in BALB/c mice orthotopically injected with PB or PB‐PI3KC2*α* 4T1 (right panels). Representative H&E staining is shown (c) (scale bar = 2 mm). Each dot in the graph (d) is representative of an injected mouse (n ≥ 5). e) Real‐time analysis performed on PB or PB‐PI3KC2*α* MCF7. mRNA levels of indicated genes are reported as ratio over control. f) PB or PB‐PI3KC2*α* MCF7 were prepared and cultured on glass coverslips covered with cross‐linked gelatin overlaid with Oregon GreenTM 488. Cells were stained with phalloidin to identify actin filaments (F‐actin; red) and ToPRO3 (nuclei, blue). Degraded gelatin is identified as a dark area (white arrows) on the Oregon GreenTM 488 (green) background. g) Representative pictures and quantification of Transwell invasion assay in PB and PB‐PI3KC2*α* MCF‐7; scale bar = 100 µm. h) Immunofluorescence staining using Phalloidin to detect F‐actin in PB or PB‐PI3KC2*α* MCF‐7. Extensive filopodia formation can be observed in PB‐PI3KC2*α* group as indicated by red arrow. i) Frequency distribution showing filopodia number in PB or PB‐PI3KC2*α* MCF7. n = 162 and n = 168 cells were imaged for PB and PB‐PI3KC2*α* MCF7, respectively, in three independent experiments. j) Filopodia length measured in PB or PB‐PI3KC2*α* MCF7 cells. n = 46 and n = 51 cells were imaged for PB and PB‐PI3KC2*α* MCF7, respectively, in three independent experiments. k,l) Representative cell tracks over 10 h (k) and quantification of the migration distance and speed (l) are shown. Data were analyzed using Manual Tracking plugin (ImageJ). n = 34 and n = 35 cells for PB and PB‐PI3KC2*α* respectively. m) Representative pictures and quantification of Transwell migration assay in PB and PB‐PI3KC2*α* MCF‐7; scale bar = 100 µm. All results are shown as mean of at least three independent experiments ± SEM (n.s., no significance, *P<0.05; **P<0.01; ***P<0.001).

We previously reported that reduced expression of PI3KC2*α* in a cohort of more than 2000 breast cancer patients initially causes impairment of tumor growth but later leads to the convergent evolution of fast‐growing clones with mitotic checkpoint defects.^[^ ^12^ ^]^ Although we first focused on patients with low PI3KC2*α* expression levels, a large subset of cases from the same cohort showed increased PI3KC2*α* expression, correlating with an enhanced probability of distant‐metastatic events.^[^ ^12^ ^]^ In particular, we extracted from our previous analysis^[^ ^12^ ^]^ the low‐grade (1–2) and high‐grade tumors (grade‐3) of patients expressing either low or high levels of PI3KC2*α* and represented these data in a pie chart in **Figure** **1**a. We observed that in patients with reduced PI3KC2*α* expression, only 26% of the tumors were scored as grade‐3 (Figure 1a, upper panel and **Table** **1**; *p*<0.00001), in agreement with our findings showing that reduced PI3KC2*α* is initially protective in breast cancer.^[^ ^12^ ^]^ In contrast, in the subset of patients expressing high levels of PI3KC2*α*, 49% of tumors were scored as grade 3 (Figure 1a, lower panels; Table 1; odds ratio, 3.03; 95% confidence interval (CI), 2.47–3.71; *p*<0.00001), suggesting that enhanced PI3KC2*α* expression was linked to increased aggressiveness and metastatic spreading.^[^ ^12^ ^]^

**Table 1:** Association between PI3KC2*α* status and tumor grade

	Grade 1–2	Grade 3	Column Totals	Odds Ratio	95% CI	Head 5 [units]
PI3KC2*α* low n (% row)	627 (74%)	216 (26%)	843	3.03	2.47 to 3.71	*P* < 0.00001
PI3KC2*α* high n (% row)	442 (51%)	462 (49%)	904			
Row Totals	1069	678	1747			

We therefore analyzed PI3KC2*α* protein expression in different human and murine breast cancer cell lines. Increased PI3KC2*α* protein expression was observed in cells with higher invasive ability^[^ ^13^ ^]^ (Figure 1b). Accordingly, analysis of PIK3C2A mRNA levels from a Cancer Cell Line Encyclopedia (CCLE) panel^[^ ^14^ ^]^ in luminal (low aggressiveness) or basal‐like/triple‐negative (highly aggressive) human breast cancer cell lines showed a significant correlation between high PIK3C2A expression and basal‐like/triple‐negative subtype (Figure S1a, Supporting Information).

To study whether increased PI3KC2*α* expression was sufficient to induce tumor metastasis in vivo, we focus on two murine breast cancer cell lines, 168‐FARN and 4T1, respectively with low and high metastatic ability (Figure 1b).^[^ ^15^ ^]^ These two cell lines were engineered with a PiggyBac (PB) transposon vector to yield stable overexpression of PI3KC2*α* (Figure S1b, Supporting Information) and orthotopically injected into the mammary fat pad in mice.^[^ ^16^ ^]^ Although increased PI3KC2*α* levels did not induce significant changes in tumor growth (Figure S1c–l, Supporting Information), overexpression of PI3KC2*α* resulted in a significantly increased number of lung metastases (Figure 1c, d). This effect was particularly evident in cells with low metastatic ability such as 168‐FARN, where enhanced PI3KC2*α* expression led to a more than fivefold increase of lung metastases (Figure 1c, d).

To better understand the molecular mechanism by which PI3KC2*α* overexpression promotes metastasis, human breast cancer cell lines expressing low levels of PI3KC2*α*, like MCF7 and MDA‐MB‐468, were engineered with a PiggyBac (PB) transposomal vector to produce a stable overexpression of PI3KC2*α* (Figure S1m, Supporting Information). After 7 days in culture, differential gene expression associated with increased cell migration and invasion was observed in MCF7 and MDA‐MB‐468 overexpressing PI3KC2*α* (PB‐PI3KC2*α*), compared to control cells (PB empty vector) (Figure 1e and Figure S1n, Supporting Information). Accordingly, increased extracellular matrix degradation was observed in PB‐PI3KC2*α* compared to control PB cells in both gelatin degradation assay and transwell invasion assays (Figure 1f,g and Figure S2a,b, Supporting Information). No significant differences in cell proliferation were observed in PB‐PI3KC2*α* compared to PB cells (Figure S1o,p, Supporting Information).

Enhanced cell invasion is accompanied by changes in the actin cytoskeleton towards a pro‐migratory phenotype.^[^ ^17^ ^]^ To better understand if PI3KC2*α* overexpression triggers rearrangements in cell shape, that is, increased number of actin protrusions, such as filopodia and lamellipodia, PB and PB‐PI3KC2*α* MCF7 were stained for F‐actin. Breast cancer cells overexpressing PI3KC2*α* exhibited more stress fibers and intense ruffling at the cell edges, together with increased numbers and length of filopodia without significant alteration in the cell area (Figure 1h–j, Figure S2c,d, Supporting Information). Spreading of filopodia and lamellipodia in PB‐PI3KC2*α* cells was even more obvious when cells were cultured on gelatin‐coated plates (Figure S2e, Supporting Information). In line with actin cytoskeleton remodeling and increased levels of pro‐migratory genes, PI3KC2*α*‐overexpression increased the migration velocity by threefold and doubled the distance traveled by breast cancer cells in a cell‐tracking migration assay (Figure 1k,l). Wound healing and transwell migration assays further confirmed that PI3KC2*α* overexpression enhances migration in both human (MCF7 and MDA‐MB‐468) and murine (4T1) breast cancer cell lines (Figure 1m and Figure S2f–i, Supporting Information). Consistent with the previous report,^[^ ^18^ ^]^ PI3KC2*α* knockdown impairs migration/invasion ability in MDA‐MB‐231 (Figure S2j, Supporting Information).

Taken together, our findings demonstrate that PI3KC2*α* promotes breast cancer cell migration and invasion in vitro and its increased expression correlates with breast cancer metastasis in vivo.

### 2.2. Increased PI3KC2*α* Expression Reduced Focal Adhesion Number and Stability

**Figure 2:**
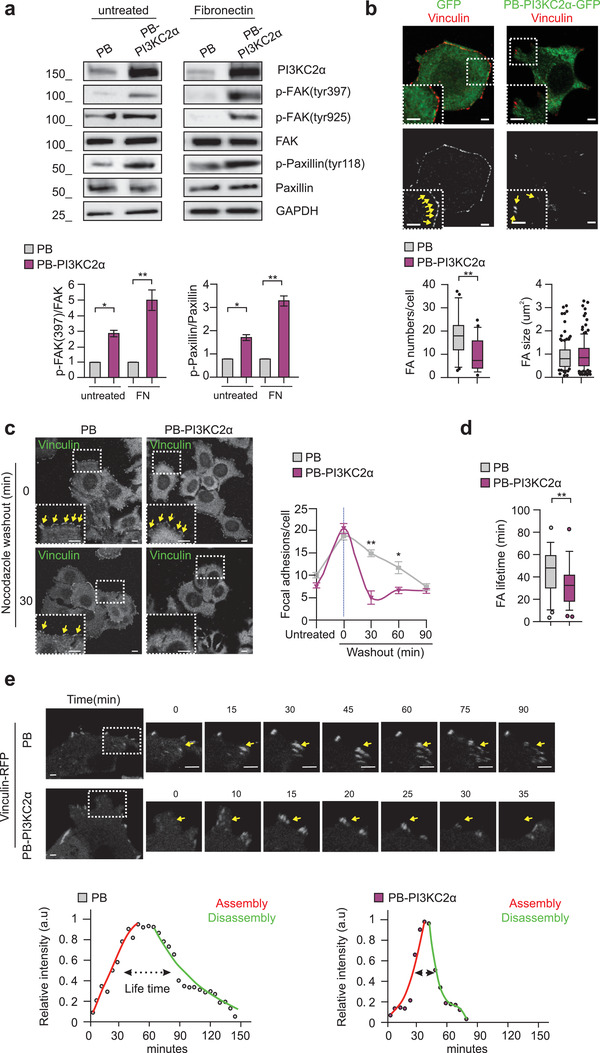
Increased PI3KC2*α* expression enhanced focal adhesion disassembly rate. a) Immunoblot analysis (upper panel) and p‐FAK(tyr397) and p‐Paxillin(tyr118) protein quantification (lower panels) performed on untreated or Fibronectin treated PB or PB‐PI3KC2*α* MCF7. b) Immunofluorescence performed on PB‐GFP and PB‐PI3KC2*α*‐GFP MCF7 showing Vinculin (red) and GFP (green). Quantification of focal adhesions number and size is shown in the Tukey box plots below. n = 12 cells and n = 21 for PB‐GFP or PB‐PI3KC2*α*‐GFP respectively from three independent experiments. c) Nocodazole washout assay was used to assess focal adhesion disassembly time (see Experimental Section). PB and PB‐PI3KC2*α* MCF7 were fixed at indicated time points and immunostained for Vinculin. Representative pictures (left) and quantification of focal adhesions number per cell are shown (right). At least 6 images per time point were analyzed (5–10 cells per picture). Scale bar, 10 µm. d,e) Quantification (d) of focal adhesion lifetime from time‐lapse performed on living cells expressing Vinculin‐RFP (e) PB or PB‐PI3KC2*α* MCF7. n ≥ 23 focal adhesions from three independent experiments. The red and green lines are respectively a logistic fit of the assembly and an exponential fit of the disassembly phase. Adhesions lifetimes are indicated by dashed arrows as defined by fluorescence intensity above the half maxim of the fit. All results are shown as mean of at least three independent experiments ± SEM (n.s., no significance, *P<0.05; **P<0.01).

Cell migration requires the integration and coordination of specific focal adhesion dynamics at the cell front, center, and rear.^[^ ^2^, ^19^ ^]^ The regulation of adhesion turnover and disassembly involves a number of tyrosine kinases and phosphatases, most of which are engaged in FAK signaling pathways.^[^ ^2a^ ^]^ To investigate the mechanisms underlying the PI3KC2*α*‐dependent cell migration in breast cancer cells, the relationship between PI3KC2*α* and focal adhesion dynamics was further explored. First, we analyzed the phosphorylation status of FAK in cells overexpressing PI3KC2*α* in normal or pro‐migratory conditions including treatment with fibronectin. We observed in PB‐PI3KC2*α* cells that phosphorylation at Tyr397, the most important site for the regulation of FAK activity,^[^ ^20^ ^]^ was significantly increased (**Figure** **2**a and Figure S3a, Supporting Information), together with enhanced phosphorylation at Tyr925 (Figure 2a and Figure S3a, Supporting Information), which is known to regulate cross‐talk between focal adhesion turnover and cell protrusion.^[^ ^21^ ^]^ In addition, PB‐PI3KC2*α* cells exhibited increased phosphorylation of the focal adhesion scaffold paxillin on Tyr118 (Figure 2a and Figure S3a, Supporting Information), a phosphorylation status commonly observed in metastatic breast cancer.^[^ ^22^ ^]^

Phosphorylation of FAK at Tyr‐397 promotes cell migration by inducing disassembly of focal adhesions at the cell tail.^[^ ^23^ ^]^ In line with this, breast cancer cells with PI3KC2*α* overexpression and increased phosphorylation of Tyr‐397 displayed a significantly reduced number of focal adhesions without changes in size (Figure 2b), suggesting rapid focal adhesion turnover. To test this directly, we performed a focal adhesion disassembly assay^[^ ^24^ ^]^ and measured the focal adhesion lifetime. Nocodazole treatment was used to induce focal adhesion accumulation followed by nocodazole wash‐out to measure the disassembly rate. At 30 min after release from the nocodazole block, PB‐PI3KC2*α* MCF7 showed more than 60% of focal adhesion disassembly, compared with less than 20% in PB control cells (Figure 2c), indicating faster disassembly of focal adhesions. Similar results were observed in MDA‐MB‐468 overexpressing PI3KC2*α* (Figure S3b, Supporting Information). Finally, time‐lapse analysis of living cells expressing Vinculin‐RFP was performed to measure the stability of focal adhesions over time. Analysis of focal adhesion lifetime showed reduced focal adhesion stability and faster focal adhesion turnover in PB‐PI3KC2*α* cells compared to PB controls (Figure 2d, e).

Collectively, our findings demonstrate that overexpression of PI3KC2*α* promotes activation of the FAK signaling pathway and shortens focal adhesion lifetime by facilitating their disassembly.

### 2.3. PI3KC2*α* is Recruited to the Focal Adhesion by the HBD Region and Produces PI(3,4)P2

**Figure 3:**
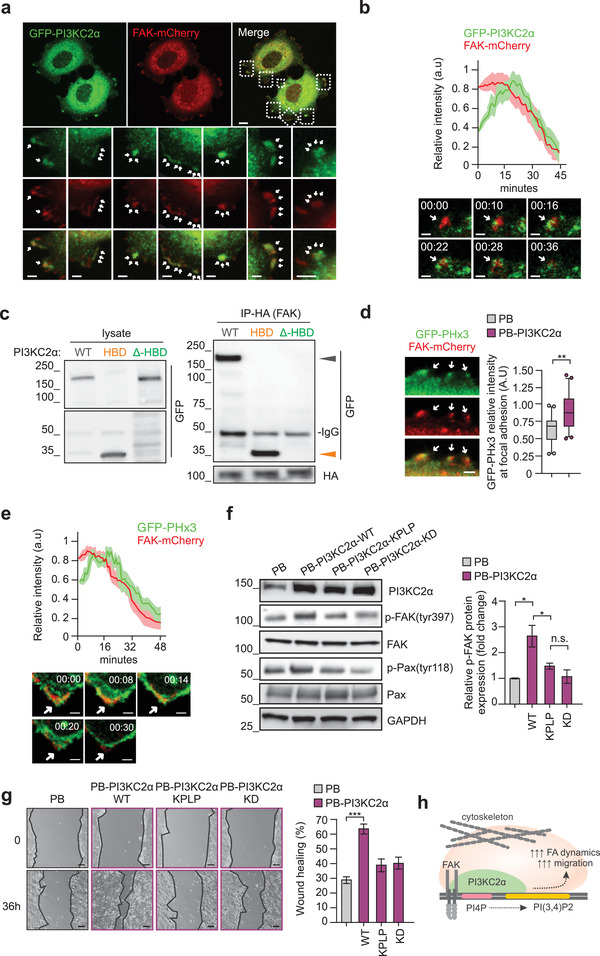
Increased PI3KC2*α* expression enhanced focal adhesion disassembly rate. a) Live cell imaging performed in MCF7 expressing FAK‐mCherry (red) and GFP‐PI3KC2*α* (green). Enlarged sections show co‐localization between PI3KC2*α* and FAK. b) Turnover dynamics of FAK‐mCherry labeled focal adhesions (red) and GFP‐PI3KC2*α* (green). Fluorescence intensity profiles measured as a function of time were normalized to the maximum FAK‐mCherry fluorescence intensity for each focal adhesion and aligned relative to one another (n = 13 focal adhesions). c) HEK293T cells were transfected with HA‐FAK and GFP‐PI3KC2a (WT, HBD or HBD deleted mutants). HA‐FAK was immunoprecipitated with anti HA antibody (IP). Bound proteins were blotted with anti GFP or anti HA. d) Representative picture (left) and PI(3,4)P2 quantification at focal adhesion (right) in live cell imaging performed on MCF7 expressing GFP‐TAPP1‐PHx3 (green) and FAK‐mCherry (red). n = 44 and n = 63 FAs for PB or PB‐PI3KC2*α* respectively. Scale bar, 1 µm. e) Turnover dynamics of FAK‐mCherry labeled focal adhesions (red) and GFP‐TAPP1‐PHx3 (green). Fluorescence intensity profiles measured as a function of time were normalized to the maximum FAK‐mCherry fluorescence intensity for each focal adhesion and aligned relative to one another (n = 10 focal adhesions). f) Immunoblot analysis (left panel) and p‐FAK(tyr397) and p‐Paxillin(tyr118) protein quantification (right panel) performed on untreated PB or PB‐PI3KC2*α*‐WT, PB‐PI3KC2*α*‐KPLP (PI3P‐producing only), or PB‐PI3KC2*α*‐KD (kinase inactive) MCF7. g) Wound healing assay performed on PB, PB‐PI3KC2*α*‐WT, PB‐PI3KC2*α*‐KPLP, or PB‐PI3KC2*α*‐KD MCF7. Representative pictures are shown on left panel and quantification of the wound area closure on right panel. Scale bar, 100 µm. h) Schematic representation showing that PI3KC2*α* is recruited to focal adhesion through association with FAK by its HBD region during focal adhesion turnover. Here PI3KC2*α* synthetizes PI(3,4)P2 required for FAK/paxillin activation and cell migration. All results are shown as mean of at least three independent experiments ± SEM (n.s., no significance, *P<0.05; **P<0.01).

To better understand how PI3KC2*α* overexpression affects focal adhesion dynamics, its intracellular localization was analyzed. Co‐expression of GFP‐PI3KC2*α* and FAK‐mCherry in MCF7 showed that the two proteins are enriched and co‐localized at plasma membrane regions corresponding to lamellipodia and filopodia (**Figure** **3**a). Time‐lapse imaging further revealed that GFP‐PI3KC2*α* enrichment precedes the disassembly of the focal adhesion itself as FAK‐mCherry decreased immediately following the full recruitment of GFP‐PI3KC2*α* (Figure 3b and Figure S4a, Supporting Information). Next, we asked how PI3KC2*α* is recruited to focal adhesions. We took advantage of our recently described crystal structure of PI3KC2*α* in which we identified a sequence insertion of about 100 amino acids between the RBD and the N‐C2 domain that was absent in class I and class III enzymes. This domain, named HBD, displayed structural similarity with the focal‐adhesion targeting (FAT) domain of Crk‐associated substrate (Cas), and the F‐actin binding domains of vinculin and *α*‐catenin. We posited that this protein‐binding domain may be responsible for the recruitment of PI3KC2*α* to focal adhesions. To test this hypothesis, 293T cells were transfected with GFP‐PI3KC2*α* (full length), GFP‐HBD, or GFP‐PI3KC2*α*ΔHBD together with HA‐FAK. Co‐immunoprecipitation (IP) experiments showed that full‐length PI3KC2*α* or HBD alone could both interact with HA‐FAK (Figure 3c and Figure S4b, Supporting Information). Conversely, HBD deletion from full‐length PI3KC2*α* completely abolished the binding to FAK (Figure 3c). Consistently, live‐cell imaging showed that GFP‐HBD co‐localizes with Vinculin‐RFP, while deletion of the HBD region from full‐length PI3KC2*α* was sufficient to displace its localization from focal adhesions (Figure S4c, Supporting Information). Taken together, our data demonstrate that the localization of PI3KC2*α* to focal adhesions is mediated by a previously unidentified association of PI3KC2*α* with FAK via its unique HBD domain.

Once recruited to the plasma membrane, PI3KC2*α* undergoes a conformational change towards an active state able to synthetize PI(3,4)P2.^[^ ^25^ ^]^ We hypothesized that the FAK‐mediated recruitment of PI3KC2*α* close to the plasma membrane could be responsible for the production of a selected PI(3,4)P2 pool leading to focal adhesion reduced stability. We generated breast cancer cells that co‐express a GFP‐TAPP1‐PHx3 binding probe able to detect PI(3,4)P2^[^ ^26^ ^]^ and FAK‐mCherry. PI(3,4)P2 showed significant co‐localization with FAK and was particularly enriched at focal adhesions where PI3KC2*α* was overexpressed (Figure 3d). Time‐lapse analysis further showed that PI(3,4)P2 is enriched at focal adhesions immediately before focal adhesion dismantling (Figure 3e), suggesting a causal role in controlling focal adhesion lifetime. To investigate whether PI(3,4)P2 directly stimulates focal adhesion turnover, we analyzed the phosphorylation status of FAK and paxillin in cells overexpressing wild type (WT), kinase‐inactive (KD), and KPLP‐mutant PI3KC2*α*. Increased phosphorylation of FAK (p‐Tyr397) and paxillin (p‐Tyr118) was observed in cells overexpressing WT PI3KC2*α* but not in KD or KPLP mutants, demonstrating that focal adhesion turnover specifically requires PI(3,4)P2 (Figure 3f). Finally, the migratory ability of cells overexpressing WT, KD, and KPLP‐mutant was assessed in a wound‐healing assay. In agreement with reduced FAK and paxillin activation, wound closure in KD‐ and KPLP‐expressing cells was slower than in WT controls (Figure 3g).

These results demonstrate that recruitment of PI3KC2*α* by its HBD region to the focal adhesion is required for PI(3,4)P2‐dependent FAK/paxillin activation and cell migration (Figure 3h).

### 2.4. PI3KC2*α*‐Dependent PI(3,4)P2 Controls R‐RAS Inactivation at Focal Adhesion

**Figure 4:**
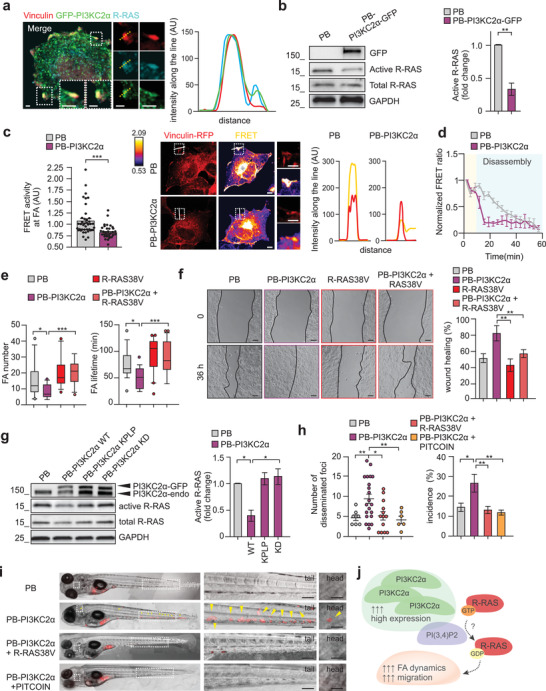
PI3KC2*α*‐dependent PI(3,4)P2 controls R‐RAS inactivation at focal adhesion. a) Immunofluorescence staining performed on MCF7 showing colocalization of GFP‐PI3KC2*α* (green) with endogenous R‐RAS (cyan) and Vinculin (red). Fluorescence intensity along the dashed line is quantified on right panel. b) Pull‐down assay (left) and quantification (right) showing active R‐RAS in PB or PB‐PI3KC2*α* MCF7. c) PB and PB‐PI3KC2*α* COS7 cells transiently expressing Raichu‐R‐RAS FRET probe showing R‐RAS activity (higher FRET ratio) at the focal adhesion stained by Vinculin‐RFP. FRET ratio at focal adhesion is quantified by using ImageJ software (left panel). Representative FRET ratio images are shown together with intensity along the line (right panels). n = 40 and n = 32 focal adhesions for PB or PB‐PI3KC2*α* respectively. Scale bar = 10 µm. d) Time‐lapse analysis of normalized R‐RAS FRET ratio at focal adhesions during their disassembly performed in PB or PB‐PI3KC2*α* MCF7 expressing Vinculin‐RFP and Raichu‐R‐RAS FRET probe. The resulting time course was normalized by dividing the ratio at each time point by the basal value at time zero, n ≥ 10 focal adhesions. e) Quantification of focal adhesion numbers and lifetime from time‐lapse performed on living MCF7 cells expressing Vinculin‐RFP and PB, PB‐PI3KC2*α*, R‐RAS 38V, or PB‐PI3KC2*α* + R‐RAS 38V respectively. n ≥ 10 focal adhesions from three independent experiments. f) Wound healing assay performed on PB, PB‐PI3KC2*α*, R‐RAS 38V, or PB‐PI3KC2*α* + R‐RAS 38V MCF7. Representative pictures are shown on left panels and quantification of the wound area closure on right panel. Scale bar, 100 µm. g) Pull‐down assay (left) and quantification (right) showing active R‐RAS in PB, PB‐PI3KC2*α*‐WT, PB‐PI3KC2*α*‐KPLP, or PB‐PI3KC2*α*‐KD MCF7. h,i) Quantification (h) and representative pictures (i) showing the number of disseminated foci and incidence of metastases in zebrafish injected with PB, PB‐PI3KC2*α*, PB‐PI3KC2*α* + R‐RAS 38V, or PB‐PI3KC2*α* + PITCOIN MCF7. Arrows indicate metastatic foci in zebrafish tail and head. n = 27 (PB), n = 77 (PB‐PI3KC2*α*), n = 94 (PB‐PI3KC2*α* + R‐RAS 38V) and n = 42 (PB‐PI3KC2*α* + PITCOIN) zebrafish. Scale bar = 200 or 25 µm in enlarged sections. j) Schematic representation showing that PI3KC2*α* overexpression reduces R‐RAS activation due to PI(3,4)P2 production at focal adhesion. This results in increased focal adhesion disassembly rate and enhanced cell migration both in vitro and in vivo. All results are shown as mean of at least three independent experiments ± SEM (n.s., no significance, *P<0.05; **P<0.01; ***P<0.001).

PI(3,4)P2 has recently emerged as a crucial regulator of cell migration in Dictyostelium, where it recruits two PH‐domain containing RasGAP proteins,^[^ ^27^ ^]^ suggesting that a RAS isoform and RasGAP pair may regulate mammalian cell migration in a similar manner. In particular, alteration in R‐RAS activity leads to changes in epithelial cell motility and morphology.^[^ ^28^ ^]^ Thus, we determined the intracellular localization of PI3KC2*α* and R‐RAS in breast cancer cells. Significant co‐localization between GFP‐PI3KC2*α* and R‐RAS at focal adhesion was observed (**Figure** **4**a and Figure S5a, Supporting Information), suggesting a functional relationship between the two proteins. To further probe this, the R‐RAS activity was evaluated in cells overexpressing PI3KC2*α* using pull‐down assays.^[^ ^29^ ^]^ R‐RAS activity was significantly reduced in PB‐PI3KC2*α* compared with PB controls (Figure 4b), indicating that increased PI3KC2*α* expression leads to R‐RAS inactivation. Other RAS family members were unaffected (K‐, N‐, H‐RAS and RAP1) (Figure S5b, Supporting Information). To challenge our findings by an independent approach, a FRET‐based sensor was used to directly visualize the activation status of R‐RAS at focal adhesions.^[^ ^29^, ^30^ ^]^ Raichu‐R‐Ras FRET probe comprised a modified YFP designated as Venus, R‐Ras, the RA domain of the R‐Ras effector RalGDS, a modified CFP designated as SECFP. Under R‐Ras activation, the intramolecular binding of R‐Ras to the RA domain brings CFP into proximity with YFP, triggering a FRET increase. We transfected cells with RFP‐vinculin to visualize focal adhesions together with a FRET probe and FRET activity was measured specifically at vinculin‐positive regions of the cell. In line with the data from the R‐RAS pull‐down assay, FRET activity was strongly reduced at focal adhesions in PB‐PI3KC2*α* cells compared with PB controls (Figure 4c). Time‐lapse imaging further revealed that focal adhesion disassembly is accompanied by inactivation of R‐RAS and that in PB‐PI3KC2*α* cells, the R‐RAS inactivation is faster compared to PB controls (Figure 4d, Figure S5c, Supporting Information). These findings demonstrate that increased PI3KC2*α* expression leads to selective and spatiotemporal inactivation of R‐RAS at focal adhesions.

To check whether R‐RAS inactivation was linked to the reduced focal adhesion lifetime and increased migration observed in cells overexpressing PI3KC2*α*, rescue experiments using a constitutively active form of R‐RAS (R‐RAS 38V) were performed. First, we analyzed the number and stability of the focal adhesions. We observed that R‐RAS 38V expression in PB‐PI3KC2*α* cells restored focal adhesions number and lifetime (Figure 4e and Figure S5d–f, Supporting Information). Second, the expression of R‐RAS 38V fully prevented the accelerated wound healing and decreased filopodia observed in cells overexpressing PI3KC2*α* (Figure 4f and Figure S6a, Supporting Information). Furthermore, R‐RAS activity was reduced only in cells expressing WT PI3KC2*α* but not cells expressing either the KD or the KPLP PI3KC2*α* mutant, indicating that R‐RAS inactivation specifically requires a PI3KC2*α*‐dependent production of PI(3,4)P2. (Figure 4g).

Finally, to monitor motility and tissue invasiveness of PI3KC2*α*‐overexpressing breast cancer cells in vivo, we took advantage of zebrafish model, to trace metastasis by live imaging in embryos.^[^ ^31^ ^]^ Hence, we injected PB (green), PB‐PI3KC2*α* (red) in 48‐hpf zebrafish (Figure S6b, Supporting Information). As expected, we observed an increased incidence of metastases and of disseminated foci in fish injected with PB‐PI3KC2*α* breast cancer cells (Figure 4h, i and Figure S6b, Supporting Information). Conversely, overexpression of R‐RAS 38V in PB‐PI3KC2*α* cells or treating PB‐PI3KC2*α* cells with PI3KC2*α* selective inhibitor, PITCOIN was sufficient to block cell spreading (Figure 4h, i).

Collectively our data point to a specific requirement of PI(3,4)P2 at focal adhesions to coordinate R‐RAS activity, focal adhesion stability, and cell migration (Figure 4j).

### 2.5. R‐RAS Inactivation is Mediated by PI(3,4)P2‐Dependent RASA3 Accumulation at Focal Adhesions

**Figure 5:**
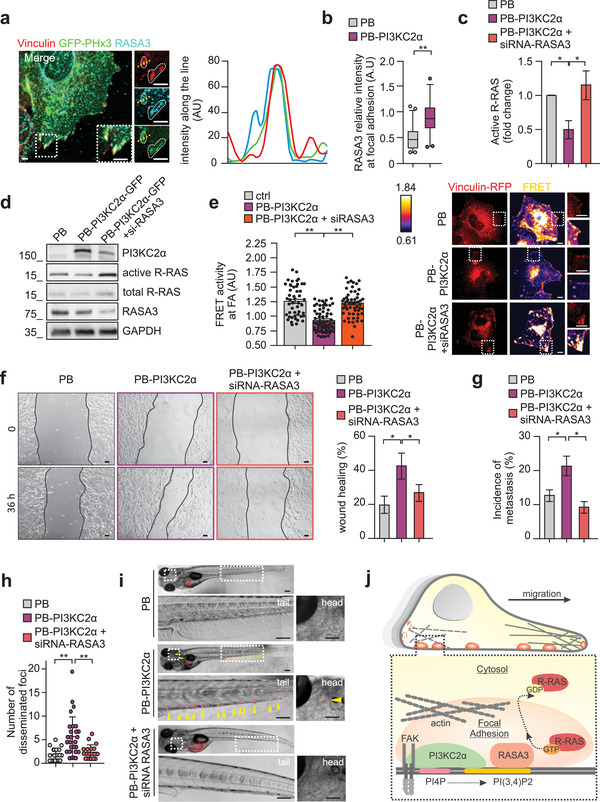
R‐RAS inactivation is mediated by PI(3,4)P2‐dependent RASA3 accumulation at FAs. a) Immunofluorescence staining performed on MCF7 showing colocalization of GFP‐TAPP1‐PHx3 (green) with endogenous RASA3 (cyan) and Vinculin (red). Fluorescence intensity along the dashed line is quantified on right panel. b) Immunofluorescence quantification of RASA3 intensity at focal adhesion in PB or PB‐PI3KC2*α* MCF7 (n = 44 and n = 63 focal adhesions for PB or PB‐ PI3KC2*α* respectively). c,d) Quantification (c) of pull‐down assay (d) showing active R‐RAS in PB, PB‐PI3KC2*α*, and PB‐PI3KC2*α* + siRNA‐RASA3 MCF7. e) PB, PB‐PI3KC2*α*, and PB‐PI3KC2*α* + siRNA RASA3 COS7 cells transiently expressing Raichu‐R‐RAS FRET probe showing R‐RAS activity (higher FRET ratio) at the focal adhesion stained by Vinculin‐RFP. FRET ratio at focal adhesion is quantified by using ImageJ software (left panel). Representative FRET ratio images are shown (right panels). n = 59 (PB), n = 82 (PB‐PI3KC2*α*), and n = 60 (PB‐PI3KC2*α* + siRNA‐RASA3) focal adhesions. Scale bar = 10 µm. f) Wound healing assay performed on PB, PB‐PI3KC2*α*, or PB‐PI3KC2*α* + siRNA‐RASA3 MCF7. Representative pictures are shown on the left panels and quantification of the wound area closure on right panel. Scale bar, 100 µm. g–i) Quantification (g, h) and representative pictures (i) showing the incidence of metastases (g) and number of disseminated foci (h) in zebrafish injected with PB, PB‐PI3KC2*α*, and PB‐PI3KC2*α* + siRNA‐RASA3 MCF7. Arrows indicate metastatic foci in zebrafish tail and head (i). n = 81 (PB), n = 87 (PB‐PI3KC2*α*), and 94 (PB‐PI3KC2*α* + siRNA‐RASA3) zebrafish. Scale bar = 200 µm or 25 µm in enlarged sections. j) Schematic representation showing that PI3KC2*α* produces PI(3,4)P2 at focal adhesion which recruits RASA3 that in turn inactivates R‐RAS. This results in increased focal adhesion disassembly rate and enhanced cell migration both in vitro and in vivo. All results are shown as mean of at least three independent experiments ± SEM (n.s., no significance, *P<0.05; **P<0.01).

The ability of R‐RAS to switch between the active and inactive state is regulated by the balance between GEFs and GAPs activity.^[^ ^32^ ^]^ Our observation of reduced R‐RAS activity points to a selective recruitment of a RasGAP able to interact with PI(3,4)P2 at focal adhesions. A potential candidate is the RasGAP RASA3, which constitutes a component of the PI(3,4)P2 interactome.^[^ ^33^ ^]^ Localization of RASA3 was then analyzed by immunofluorescence in breast cancer cells. Significant co‐localization between RASA3 and PI(3,4)P2 (stained with GFP‐TAPP1‐PHx3 probe) was observed at vinculin‐positive focal adhesions (**Figure** **5**a and Figure S6c, Supporting Information). Accordingly, cells overexpressing PI3KC2*α* with increased PI(3,4)P2 levels showed a twofold increase in RASA3 enrichment at focal adhesions (Figure 5b and Figure S6d, Supporting Information). Coherently, RASA3 knockdown in cells overexpressing PI3KC2*α* restored R‐RAS activation, demonstrating a functional relationship (Figure 5c, d).

To link the recruitment of RASA3 at focal adhesions with its GAP activity towards R‐RAS, we performed a FRET analysis to measure R‐RAS activation. While in PB‐PI3KC2*α* cells, R‐RAS activity was significantly reduced at focal adhesions, knock‐down of RASA3 was sufficient to restore R‐RAS activity to levels comparable to control PB cells (Figure 5e). Therefore, RASA3 controls R‐RAS activation at focal adhesion in a PI(3,4)P2‐dependent manner. Then we checked whether RASA3‐mediated inactivation of R‐RAS directly affects cell migration in wound healing assays. While PB‐PI3KC2*α* cells showed a fast closure of the wound, concomitant down‐modulation of RASA3 was sufficient to slow the wound closure similar to the rate observed in control cells (Figure 5f).

Finally, to challenge our findings in an in vivo model, we used an in vivo zebrafish model.^[^ ^31^ ^]^ To this aim, we injected MCF7 expressing either PB, PB‐PI3KC2*α*, or PB‐PI3KC2*α* together with siRNA targeting RASA3 in 48‐hpf zebrafish. As expected, we observed an increased number of metastases and disseminated foci in fish injected with PB‐PI3KC2*α* breast cancer cells. Conversely, knocking‐down of RASA3 in PB‐PI3KC2*α* cells was sufficient to block the migratory process (Figure 5g–i).

Collectively, our findings demonstrate that PI(3,4)P2‐RASA3 mediated inactivation of R‐RAS allows PI3KC2*α*‐overexpressing breast cancer cells to acquire a pro‐migratory and pro‐invasive phenotype, leading to increased metastatic potential (Figure 5j). Moreover, we provide a proof of concept that inhibiting PI3KC2*α* or targeting RASA3 activity can block the metastatic spreading of breast cancer cells overexpressing PI3KC2*α* in vivo.

## 3 Discussion

By producing PI(3)P and PI(3,4)P2, PI3KC2*α* plays an essential role during development and its loss or inactivation leads to multiple pathological processes.^[^ ^34^ ^]^ Besides its enzymatic activity, we recently described a scaffold function of PI3KC2*α* that is necessary for keeping genome integrity by preventing mitotic defects in breast cancer cells.^[^ ^12^ ^]^ Accordingly, loss of PI3KC2*α* in breast cancer initially delays tumor growth but finally leads to the selection of more aggressive clones. Here we describe that, unlike patients with PI3KC2*α* loss, high levels of PI3KC2*α* correlate with tumor grade and probability of distant metastatic events in breast cancer patients. In agreement with this phenotype, we found that a PI(3,4)P2 pool synthetized by PI3KC2*α* at focal adhesions disturbs their stability, leading to enhanced breast cancer cell migration and invasion. Moreover, differential expression of genes involved in migration and invasion can be likely explained as a consequence of increased focal adhesion dynamics and cytoskeletal remodeling,^[^ ^35^ ^]^ induced by PI3KC2*α* overexpression. This is particularly evident in light of the fact that Class‐II PI3K has never been shown to function in gene transcriptional regulation.^[^ ^34c^ ^]^

Although our model of increased tumor cell migration and invasion relies on the PI3KC2*α* catalytic activity, the recently described PI3KC2*α* HBD domain emerged as a critical region for protein‐protein interaction. In particular, this domain appears pivotal to the localization of PI3KC2*α* to cytoskeletal structures, as initially we found it to localize this kinase to the mitotic spindle by binding with TACC3^[^ ^12^ ^]^ and now to target PI3KC2*α* to focal adhesions, by promoting its association with FAK. In line with these findings, the HBD shares structural similarities with the FAT domain of Cas, and the F‐actin binding domains of vinculin and *α*‐catenin, both necessary and sufficient for protein localization to the focal adhesions. Altogether these findings suggest that the HBD domain of PI3KC2*α* has a structural role as a protein‐interacting domain in driving fundamental association with different proteins, critically required to localize PI3KC2*α* activity in specific subcellular compartments.

While PI(3,4)P2 has been previously associated with increased cell migration^[^ ^10^, ^11^, ^36^ ^]^ and an inhibitory RAS‐PI(3,4)P2 feedback loop was recently proposed,^[^ ^27^ ^]^ its relevance for tumor metastases in vivo remained largely elusive. Our findings point to a direct role for PI(3,4)P2 in controlling focal adhesion stability, and identified the R‐Ras‐GAP RASA3 as the main player in driving R‐RAS inactivation during migration and invasion, both in vitro and in vivo. Based on our findings, PI(3,4)P2 produced by PI3KC2*α* recruits RASA3 at focal adhesions, leading to R‐RAS inactivation and focal adhesion dismantling. The localization of endogenous RASA3 at focal adhesion complexes is required for cytoskeleton rearrangement and cell migration, and loss of RASA3 impairs focal adhesion turnover.^[^ ^37^ ^]^ Importantly, our data shows that increased PI3KC2*α*‐mediated PI(3,4)P2 production significantly reinforces the localization of RASA3 at focal adhesions.

RASA3 is known to function as a dual GAP for R‐RAS and RAP1 small GTPases.^[^ ^38^ ^]^ Here we found that, at least in breast cancer epithelial cells, overexpression of PI3KC2*α* leads to increased PI(3,4)P2 enrichment at focal adhesion accompanied by an increased activity of RASA3 towards R‐RAS. Considering that the switch of RASA3 from RAS‐GAP to RAP‐GAP involves its PH domain,^[^ ^39^ ^]^ we hypothesize that PI(3,4)P2 might affect this conformational change by promoting RASA3 activity in an R‐RAS selective manner. Further studies are needed to better elucidate this mechanism of action.

Previous works showed that the C‐terminal region of R‐RAS contains a focal adhesion targeting signal and that targeting and activation of R‐RAS are linked processes in the formation of focal adhesion in epithelial cells.^[^ ^28a,b^ ^]^ Interestingly, only R‐RAS‐GTP is recruited to focal adhesions, enhancing both cell adhesion and cell spreading.^[^ ^28^, ^40^ ^]^ Conversely, deactivation of R‐RAS leads to its exclusion from focal adhesions and reduced focal adhesion stability.^[^ ^28a^ ^]^ In line with this, expression of dominant negative R‐RAS (41A) enhances migration persistence and membrane protrusion.^[^ ^41^ ^]^ Our data further expanded these findings, showing that in breast cancer cells, the R‐RAS inactivation and the consequent reduction in focal adhesion number and stability rely on PI(3,4)P2‐mediated recruitment of the Ras‐GAP, RASA3 to focal adhesions. We speculate that the RASA3‐driven inactivation of R‐RAS can be responsible for R‐RAS release from the focal adhesions and their consequent destabilization and enhanced turnover, leading to increased cell migration. Additionally, the zebrafish xenograft model conclusively demonstrated that the PI3KC2*α*/RASA3/R‐RAS axis controls breast cancer distant metastasis in vivo; nonetheless, future studies in mouse models will better elucidate the importance of targeting PI3KC2*α* and RASA3 in metastatic breast cancer therapy.

Despite recent advances in anticancer therapies targeting the primary mammary gland tumor, treating metastatic breast cancer has remained challenging. Therefore, the identification of new druggable pathways promoting metastasis remains an unmet medical need. The finding of the crosstalk between PI3KC2*α* and RASA3 identifies two potential therapeutic targets. On the one hand, the development of more potent and selective PI3KC2*α* inhibitors might open new therapeutic strategies for metastatic breast cancer. On the other, GAPs like RASA3 are potentially druggable and our proof‐of‐concept study provides evidence that inhibition of RASA3 GAP activity can significantly reduce metastasis of breast cancer irrespective of the subtype and likely even in other cancer types.

## 4 Experimental Section

### Human Subjects

To assess the clinical relevance of PI3KC2*α* expression to breast cancer, a series of 1779 operable breast cancer patients was analyzed, available on tissue microarray (TMA), who underwent surgery at the European Institute of Oncology (IEO) in Milan from years 1997 to 2000. Details were previously shown.^[^ ^12^ ^]^ The study was approved by the Institutional Review Board of the European Institute of Oncology (Milan, Italy) and informed consent was obtained from all subjects.

### Cell Lines

MCF7 was maintained in RPMI‐1640 medium (Gibco) supplemented with 10% fetal bovine serum (FBS) and 1% Penicillin‐Streptomycin (10 000 U mL^−1^). SKBR3, MDA‐MB‐468, T47D, BT474, BT549, MDA‐MB‐231, HEK293T, COS7, and mice mammary tumor cell lines (67NR, 168‐FARN, 4TO7, 4T1) were grown in Dulbecco's modified Eagle's medium (DMEM) with the same supplement as shown above. hTERT‐HME1 were purchased from ATCC and cultured MEGM TM (Mammary Epithelial Cell Growth Medium) BulletKit TM (Cat. CC‐3150) from Lonza/Clonetics Corporation. All cells were grown at 37 °C in the humidified incubator (Thermo scientific) with 5% CO_2_. Cell lines were purchased from ATCC (without further authentication) and were routinely tested to exclude mycoplasma contamination. To establish cell lines stably overexpressing PI3KC2*α*, a PiggyBac transposomal system was used. Plasmids were designed and purchased from VectorBuilder Inc. Helper plasmid, carrying the expression of the transposase, and the transposable plasmid, containing human PI3KC2*α* cDNA sequence, were transfected at 1:3 ratios^[^ ^42^, ^43^ ^]^ using X‐tremeGENE HP DNA Transfection Reagent (Roche) followed by 7 days Blasticidine S hydrochloride (BSD) (Sigma, St. Louis, MO, USA) selection. Stable cell lines were routinely tested by Immunoblot for PI3KC2*α* expression and taken in culture for no more than 20 passages.

### Protein Analysis

Cells and tissues were homogenized in lysis buffer (120 mm NaCl, 50 mm Tris‐HCl pH = 8, 1% Triton X‐100) supplemented with 25x protease inhibitor cocktail (Roche), 50 mm sodium fluoride, and 1 mm sodium orthovanadate. Lysates were cleared by centrifugation at 13 000 rpm for 15 min at 4 °C. Protein concentration was determined by Bradford method and supernatants were analyzed for immunoblotting with the indicated antibodies. Membranes probed with the indicated antibodies were then incubated with HRP conjugated secondary antibodies (anti mouse used 1:10 000, anti rabbit 1:5000, Sigma) and developed with enhanced chemiluminescence (ECL, BD). GAPDH or *α*‐tubulin were used for loading control as indicated above. The phosphorylation status of FAK and paxillin were normalized by their total levels. For IP assays, cells were lysed in 50  mm Tris‐HCl (pH ), 150  mm NaCl, 1% NP‐40, 1  mm EDTA, 10% glycerol, and protease and phosphatase inhibitors. 1 mg of pre‐cleared extracts were incubated with 1 µg of the indicated antibody at 4 °C on a rotating rack. After 1.5 h, 15 µL of protein G‐Sepharose (Amersham Biosciences, Buckinghamshire, UK) were added for 30 min. Samples were collected by centrifugation (13 000 rpm 1 min) and washed six‐times with lysis buffer. Bound protein complexes were then eluted by adding 30 µL Laemmli sample buffer. For pull‐down experiment, HEK293T cells homogenized in lysis buffer (120 mm NaCl, 50 mm Tris‐HCl pH = 8, 1% Triton X‐100) supplemented with 25x protease inhibitor cocktail (Roche), 50 mm sodium fluoride, and 1 mm sodium orthovanadate. Lysates were cleared by centrifugation at 13 000 rpm for 15 min at 4 °C. 1mg of cell lysate was incubated with 15 µL of recombinant protein‐coupled with glutathione Stransferase agarose (GE, Buckinghamshire, UK) for 1 h at 4 °C. Beads were washed four times with 1 mL of reaction buffer and analyzed by Immunoblotting after the addition of 30 µL of Laemmli buffer.

### Antibodies

Anti PI3KC2*α* (#22028‐1‐AP, Proteintech), anti GFP (gift from Emilia Turco, University of Turin, Italy), anti *α*‐tubulin (#2125, Cell Signaling), anti GAPDH (sc‐47724, Santa Cruz Biotechnology), anti Myc‐tag (#2276, Cell Signaling), anti FAK (#71 433, Cell Signaling), anti p‐FAK (tyr397) (#8556, Cell Signaling), anti p‐FAK (tyr925) (#3284, Cell Signaling), anti Paxillin (#2542, Cell Signaling), anti p‐Paxillin (tyr118) (#69 363, Cell Signaling), anti HA‐tag (# 26 183, Thermofisher), anti R‐RAS (#8446, Cell Signaling), anti RASA3 (#PA5‐30445,Invitrogen), anti Rap1A/Rap1B (#4938, Cell Signaling), anti RAS (#3339, Cell Signaling), and anti Vinculin (#V9131, Sigma).

### Gene Silencing and Inhibitors

Rasa3 (5’‐GCGCTTTGGGATGAAGAAT‐3’ and 5’CCTGAAGTTTGGAGATGAA‐3’) siRNAs were purchased from Horizon Discovery/Dharmacon. PI3KC2*α*‐selective PITCOIN1 inhibitor was provided by Prof. Volker Haucke and described in a seperate study in preparation (Lo WT, et al.).

### Plasmids

PI3KC2*α* and PI3KC2*α*‐GFP PiggyBac (PB) transposomal system were purchased from VectorBuilder and mutagenized to generate PB‐PI3KC2*α*‐KD and PB‐PI3KC2*α*‐KPLP by QuikChange II Site‐Directed Mutagenesis Kit (Agilent) as previously described.^[^ ^44^ ^]^ myc‐PI3KC2*α* plasmid was previously generated.^[^ ^12^ ^]^ mTagRFP‐Vinculin was a gift from Michael Davidson (Addgene plasmid # 58 030; http://n2t.net/addgene:58030; RRID: Addgene_58 030); pmCherry‐C1‐FAK‐HA was a gift from Anna Huttenlocher (Addgene plasmid # 35 039; http://n2t.net/addgene:35039; RRID:Addgene_35 039), pCGN R‐Ras 38V was a gift from Adrienne Cox (Addgene plasmid # 14 728; http://n2t.net/addgene:14728; RRID:Addgene_14 728), GST‐RBD was a gift from Martin Schwartz (Addgene plasmid # 15 247; http://n2t.net/addgene:15247; RRID:Addgene_15 247), Raf‐1 GST RBD 1–149 was a gift from Channing Der (Addgene plasmid # 13 338; http://n2t.net/addgene:13338; RRID:Addgene_13 338), pGEXTK‐Pak1 70–117 was a gift from Jonathan Chernoff (Addgene plasmid # 12 217; http://n2t.net/addgene:12217; RRID:Addgene_12 217), and GFP‐TAPP1‐PHx3 was kindly provided from Prof. Gerald R V Hammond (University of Pittsburgh). GFP‐PI3KC2*α* WT, GFP‐HBD, and GFP‐ PI3KC2*α*‐ΔHBD were provided by Prof. Volker Hauck and PI3KC2α crystal structure was described in a seperate study in preparation (Lo WT, et al.). All plasmids were tested by restriction digestion and automated DNA sequencing. Plasmids were transfected by X‐tremeGENE HP DNA Transfection Reagent (Roche Applied Science, Penzberg, Germany), according to the manufacturer's instructions.

### Quantitative RT‐PCR

Total RNA was extracted using TRIzol reagent (Invitrogen, Carlsbad, CA). cDNA was synthesized from 1000 ng of total RNA using cDNA reverse transcription kits (Applied Biosystems, Foster City, CA). Relative mRNA level was analyzed by real‐time PCR (ABI 7900HT FAST Real‐Time PCR system, Applied Biosystems, Foster City, CA) with Taqman assays, using the Universal Probe Library system (Roche Applied Science, Penzberg, Germany). 18S gene was used as housekeeping control. The primers are listed in Table S1, Supporting Information.

### Immunofluorescence

Immunofluorescence was performed by ice‐cold methanol or 4% PFA fixation of the cells followed by standard procedures.^[^ ^12^, ^34^ ^]^ Next, cells were permeabilized with either 0.1% Saponin or 0.3% Triton X‐100 for 5 min and then blocked in 2% BSA for 20 min, followed by the incubation with indicated primary antibodies for 60 min. AlexaFluors secondary antibody (Alexa 488, Alexa 568, or Alexa 633) were used 1:1000 for 45 min. Cells were stained with DAPI and examined with, Leica TCS‐II SP5 or Leica TSC‐II SP8 confocal microscope. Raw images were digitally processed only to normalize the background and enhance the contrast. Z‐stacks were acquired and processed with the Maximum Projection tool.

### Proliferation Assay

Proliferation assay was performed by using CellTiter‐Glo Luminescent Cell Viability Assay (Promega, Mannheim, Germany). Cells were seeded into 96‐well plates in octuplicate at 4×10^3^ cells/well. Absorbance was measured at the indicated time points.

### Single Cell Tracking

Transfected MCF7 cells were seeded on µ‐Slide 8 Well (ibidi, Germany) and cultured overnight. Cells were maintained at 37 °C and 5% CO_2_ and cell migration was monitored by using Leica TCS‐II SP5 confocal microscope (10x objective). Cells were imaged for 24 h every 10 min. To assess cell migration, speed, and distance, single cells were tracked by using Manual Tracking plug‐in from ImageJ.

### Zebrafish Strains and Metastasis Assay

All procedures using zebrafish (Danio Rerio) were authorized by the Ethical Committee of the University of Torino and the Italian Ministry of Health. The wild‐type fish strains Tuebingen was used. Adult fish were routinely maintained under a 14h light and 10h dark photoperiod at approximately 28 °C, bred and genotyped according to standard procedures. Eggs were generated by natural mating, and following fertilization were collected, treated, and maintained under a 12h light and 12h dark photoperiod at 28 °C. Embryos were treated with 0.003% 1‐phenyl‐2‐thiourea (PTU, #P7629, Sigma) at 24hpf to prevent the formation of melanin pigment, which could interfere with the visualization of fluorescence in the metastatic assay. Embryos and adult fish were sacrificed with a tricaine overdose. For zebrafish xenotransplantation, 48hpf wild‐type zebrafish embryos were anesthetized with 0.04 mg mL^−1^ tricaine (Sigma, St. Louis, MO, USA) before cancer cell injection. Approximately 300 Vybrant DiI(red) or Dio (green) labeled tumor cells were injected into the yolk sac of each embryo, and zebrafish were maintained in E3 medium for 1 h at 28 °C. After confirmation of a visible cell mass at the injection site, zebrafish were maintained at 30 °C for 72h in standard embryo medium (Westerfield, 1994) supplemented by 0.003% PTU, 1 g L^−1^ glucose, and 5 mmol L^−1^ L‐glutamine. Images were acquired by Zeiss Observer‐Z1 microscope (10× objective). Due to the large size of the embryos, in the representative pictures, sequential images of each embryo were acquired and composed to show the whole embryo.

### Nocodazole Wash‐Out Assay

Cells were grown on glass coverslips, starved overnight in medium containing 1% serum, and treated with 10 µm nocodazole in serum‐free medium for 4h to depolymerize microtubules, as previously described.^[^ ^24^ ^]^ Nocodazole was washed‐out with serum‐free medium and cells were incubated at 37 °C for the indicated periods of time. Subsequently, cells were fixed and prepared for immunofluorescence staining with anti Vinculin. The number of focal adhesions per cell was quantified at all time points. Images were processed after subtracting threshold levels by the Image J software.

### Focal Adhesion Assembly and Disassembly

MCF7 were transfected with FAK–mCherry and imaged by time‐lapse microscopy using Leica TSC‐II SP8 confocal microscope for at least 1 h. Images were acquired every 3 min as previously shown.^[^ ^37^, ^45^ ^]^ Regions of interest (ROI) were defined by using the imageJ ROI tool to outline individual adhesions. If the FA significantly changed size and location and moved out of the ROI over time, ROIs were redrawn to include all fluorescence. Cytoplasmic background was subtracted by using duplicated ROIs adjacent to FAs. The fluorescence intensity of FA as a function of time was evaluated from the time‐lapse series, by quantifying the intensity of pixels with the Image J software. As previously described,^[^ ^46^ ^]^ FA lifetime analysis was defined as the time during which the fluorescence intensity remained above the half‐maximum and was calculated from the assembly and disassembly curve done using the Solver function in Excel (Microsoft). The spatiotemporal correlation of focal adhesion with GFP‐TAPP1‐PHx3 or PI3KC2*α*‐GFP as a function of time was quantified based on the focal adhesion drawn regions and the intensity of GFP‐TAPP1‐PHx3 or PI3KC2*α*‐GFP was measured with the ImageJ software.

### Transwell Assay

For cell migration assay, 3 × 10^4^ cells suspended in serum‐free medium were seeded into the upper chamber of 24‐well Boyden chamber (8 µm; Corning, NY, USA), and 500  µL medium with 10% FBS was added into bottom chamber. Cell invasion assay was performed using the chambers coated with Matrigel (Corning). After 48 h, the non‐migrated/invaded cells were removed by cotton swabs and cells migrated/invaded through the membranes were fixed with 4% PFA for 20 min and stained with 0.5% crystal violet for 30 min (Sigma, St. Louis, MO, USA). Images of five random fields for each membrane were captured by microscope. Migrated/invaded cells were counted by Image J software.

### Wound Healing

Cells grown on 24 well plates were subjected to serum starvation for 24 h. An approximately 0.4–0.5 mm wound was scratched by using a fine end of 0.1–10 µL pipette tip followed by three times washes with PBS. Cells were cultured in serum‐free medium throughout the experiment to avoid cell proliferation and the wounds’ closure was measured as the reduction area of the wound at 0 and 36 h after making the wound.

### R‐RAS FRET Analysis at Focal Adhesion

Cells were transfected with Vinculin‐RFP and Raichu‐R‐RAS FRET and cultured overnight. Next, cells were fixed in 4% PFA, washed with PBS, and blocked in 2% BSA solution and imaged using a 63x objective of Leica TSC‐II SP8 confocal microscope. Fret analyses were described previously.^[^ ^47^ ^]^ In brief, images for CFP and FRET were obtained. After background subtraction was carried out, the FRET/CFP ratio was depicted using ImageJ software, and the image resulting was used to represent FRET efficiency. FRET ratio at the focal adhesion was quantified using ImageJ software. Raichu‐R‐RAS FRET probes were kindly provided by Prof. Michiyuki Matsuda, Kyoto University.^[^ ^29b^ ^]^

### Gelatin Degradation Assay

Cells were seeded on the glass coverslip coated with Oregon Green 488‐Conjugated gelatin. After 16 h, coverslips were collected and followed by immunofluorescence staining with Rhodamine Phalloidin and ToPRO‐3. Gelatin degradation areas were quantified by Image J software.

### Mice Metastatic Model

Female BALB/c mice (8 to 12 weeks old) weighing between 18 and 20 g were housed at 22 ± 5 °C in a 12h light/dark cycle and fed rodent chow and water freely. Orthotopic mammary fat pad implantation was performed as follows: 10^6^ 4T1 cells were injected (100 µL cell suspension in PBS) in the right fourth mammary gland. Tumor growth was measured every two days after day 7, and the primary tumor was removed after 14 days when it reached 300–400 mm^3^.^[^ ^48^ ^]^ Mice were monitored every two days for labored breathing and primary tumor recurrence. After two weeks, mice were sacrificed and lungs were collected for H&E staining to count lung macro‐metastasis nodules. For 168‐FARN, 10^6^ cells were injected (100 µL cell suspension in PBS) in the right fourth mammary gland. Tumor growth was measured every week until week 6 (day42). Mice were then sacrificed and lungs were collected for H&E staining to count lung macro‐metastasis nodules.^[^ ^49^ ^]^ Protein extraction from primary tumors was used to verify PI3KC2*α* overexpression efficiency. All the animal use followed institutional animal welfare guidelines and legislation, as approved by the local Animal Ethics Committee (Comitato di Bioetica e Valutazione, Torino, Italy).

### General Experimental Approaches

All of the statistical details of experiments can be found in the figure legends, in the Results and the Experimental Section, including the statistical tests used, the exact value of n, what n represents (cells, experiments) and precision measures (mean, median, SD, SEM, confidence intervals). No statistical methods were used to predetermine sample size. No samples or data points were excluded from the reported analyses. Samples were not randomized to experimental groups. Sample size was determined on the basis of the previous studies.^[^ ^12^, ^34^ ^]^ The investigators were not blinded to allocation during experiments and outcome assessment.

### Statistical Analysis

Prism software (GraphPad) was used for statistical analysis. Significance was calculated with Student t‐test and one‐ or two‐way analysis of variance tests (ANOVA) followed by Bonferroni's post hoc analysis, or Mantel Cox log‐rank test where appropriate. Values were reported as the mean ± standard error of the mean (SEM). *p*<0.05 was considered statistically significant (∗), *p*<0.01 very significant (∗∗), and extremely significant *p*<0.001 (∗∗∗).

## Conflict of Interest

E.H. is cofounder and board member of Kither Biotech, a pharmaceutical product company developing PI3K inhibitors for the treatment of respiratory diseases, not in conflict with statements made in this article. The other authors declare no conflict of interest.

## Author Contribution

F.G. and E.H. are the co‐last authors. H.L. designed and performed research, analyzed data, and wrote the manuscript; L.P., M.H., J.M., M.C.D.S., C.Z., M.F., F.N., S.P., P.P.F., P.E.P., and M.M. performed research and analyzed data; W.L. and V.H. provided critical reagents; F.G. and E.H. jointly designed research, supervised the work and wrote the manuscript. All authors contributed to the review and editing of the manuscript.

## Supporting information

Supporting Information

## Data Availability

The data that support the findings of this study are available from the corresponding author upon reasonable request.
