# Supplementary material for: Phosphoinositide Conversion Inactivates R‐RAS and Drives Metastases in Breast Cancer
Source: Adv Sci (Weinh). 2022 Jan 31;9(9):2103249. doi: 10.1002/advs.202103249 (PMC8948670; doi:10.1002/advs.202103249)
Supplement: Supplementary file 1 — Supporting Information [file ADVS-9-2103249-s001.pdf]

ADVANCED SCIENCE
Open Access

Supporting Information

for *Adv. Sci.*, DOI 10.1002/advs.202103249

Phosphoinositide Conversion Inactivates R-RAS and Drives Metastases in Breast Cancer

Huayi Li, Lorenzo Prever, Myriam Y. Hsu, Wen-Ting Lo, Jean Piero Margaria, Maria Chiara De Santis, Cristina Zanini, Marco Forni, Francesco Novelli, Salvatore Pece, Pier Paolo Di Fiore, Paolo Ettore Porporato, Miriam Martini, Hassane Belabed, Marc Nazare, Volker Haucke, Federico Gulluni and Emilio Hirsch**

ADVANCED SCIENCE
Open Access

Supporting Information

for *Adv. Sci.,* DOI: 10.1002/advs.202103249

Phosphoinositide conversion inactivates R-RAS and drives metastases in breast cancer

Huayi Li, Lorenzo Prever, Myriam Y. Hsu, Wen-Ting Lo, Jean Piero Margaria, Maria Chiara De Santis, Cristina Zanini, Marco Forni, Francesco Novelli, Salvatore Pece, Pier Paolo Di Fiore, Paolo Ettore Porporato, Miriam Martini, Hassane Belabed, Marc Nazare, Volker Haucke, Federico Gulluni and Emilio Hirsch**

Figure S1

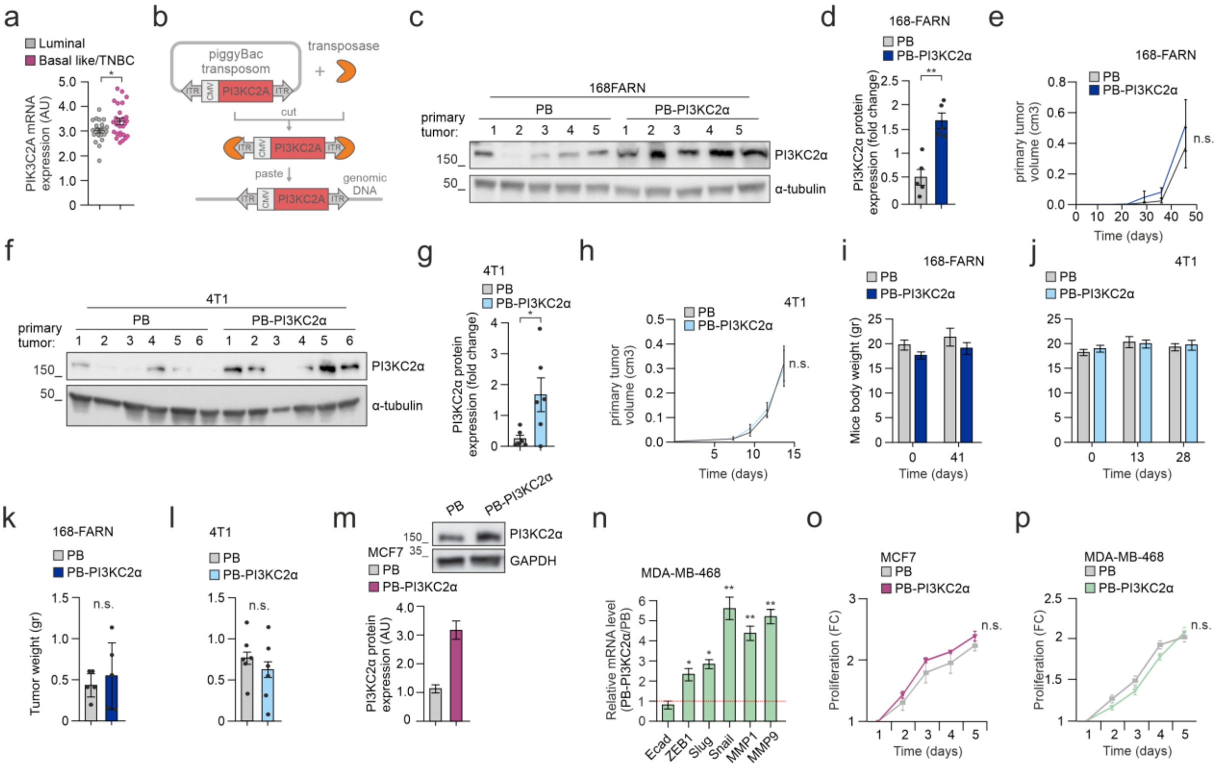

Figure S1. a) Analysis of PI3KC2α mRNA level in luminal (n = 22) and basal/triple-negative (n = 27) breast cancer cell lines available in CCLE. b) Schematic representation depicting the PiggyBac (PB) transposomal system used to produce stable overexpression of PI3KC2α. ITR: inverted terminal repeats; CMV: cytomegalovirus (CMV) immediate early enhancer and promoter. c, d) Representative immunoblots (c) and quantifications (d) showing PI3KC2α protein expression in 168-FARN derived primary tumors. Each dot in graphs (d) is representative of an injected mouse (n =5). e) Analysis of 168-FARN derived primary tumor volume (n = 5). f, g) Representative immunoblots (f) and quantifications (g) showing PI3KC2α protein expression in 4T1 derived primary tumors. Each dot in graphs (g) is representative of an injected mouse (n = 6). h) Analysis of 4T1 derived primary tumor volume (n = 6). i, j) Analysis of mice body weight in 168-FARN group (i, n = 5) and 4T1 group (f, n = 6). k, l) Analysis of primary tumor weight in 168-FARN group (i, n = 5) and 4T1 group (f, n = 6). m) Representative blots and quantification of PI3KC2α protein level in PB and PB-PI3KC2α MCF7. n) Real-time analysis performed on PB or PB-PI3KC2α MDA-MB-468. mRNA levels of indicated genes are reported as ratio over control. o, p) Proliferation analysis performed in PB or PB-PI3KC2α MCF7 (o) and PB or PB-PI3KC2α MDA-MB-468 (p). All results are shown as mean of at least three independent experiments ± SEM (n.s., no significance, *P<0.05; **P<0.01).

Figure S2

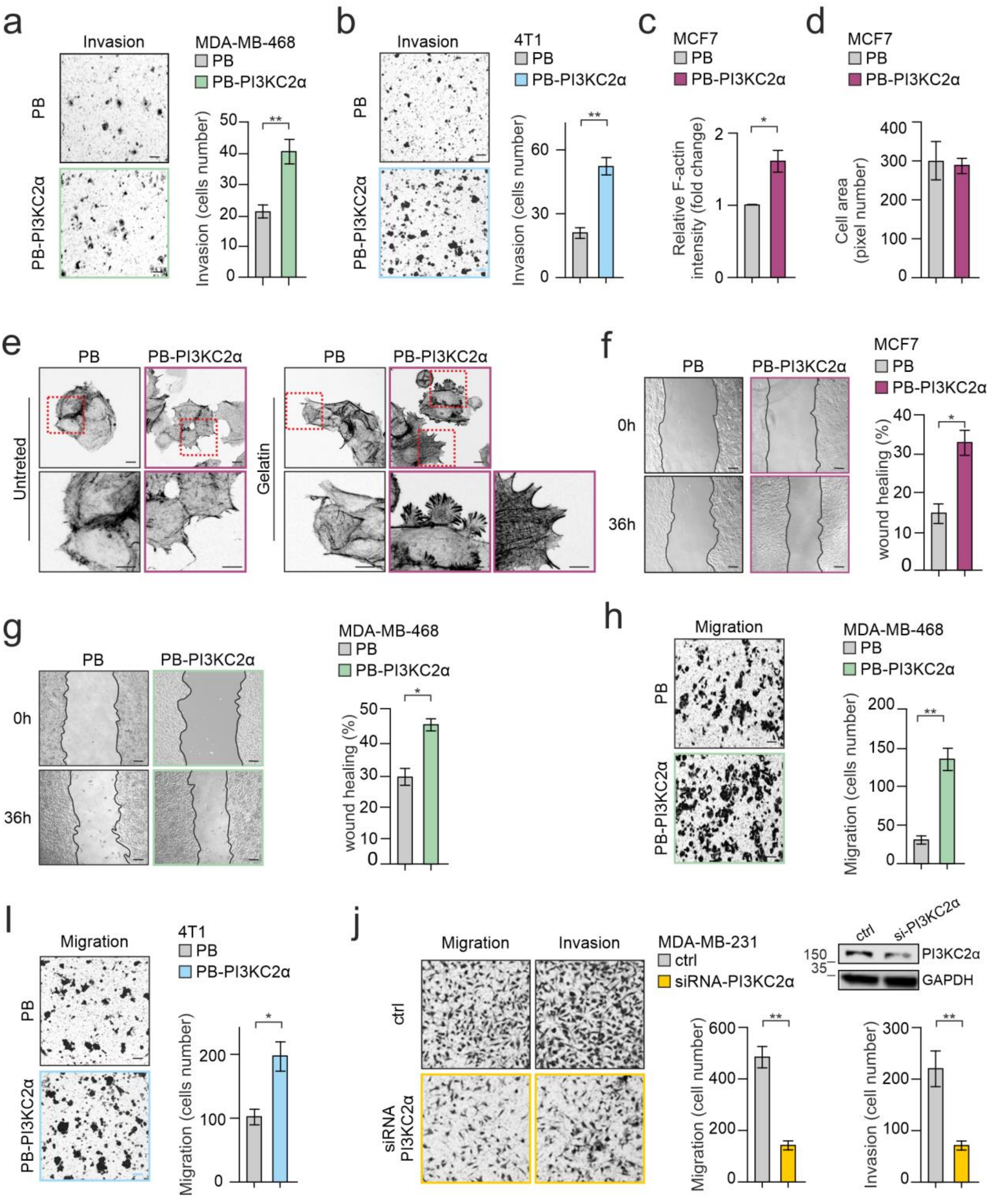

Figure S2. a, b) Transwell invasion assays performed in PB or PB-PI3KC2α MDA-MB-468(a) and PB or PB-PI3KC2α 4T1(b). Scale bar = 100 μm. c) F-actin intensity measured in PB or PB-PI3KC2α MCF7 cells. n = 46 and n = 51 cells were imaged for PB and PB-PI3KC2α MCF7, respectively, in three independent experiments. d) Cell area measured in PB or PB-PI3KC2α MCF7 cells stained for Phalloidin. n = 46 and n = 51 cells were imaged for

PB and PB-PI3KC2α MCF7, respectively, in three independent experiments. e) Immunofluorescence staining using Phalloidin to detect F-actin in PB or PB-PI3KC2α MCF-7 with or without gelatin coating. Filopodia formation can be observed in the enlarged sections. f, g) Wound healing assay performed in PB or PB-PI3KC2α MCF-7 (f) and PB or PB-PI3KC2α MDA-MB-468(g). Representative pictures (left panel) and relative quantification (right panel) are shown. Scale bar = 100 μm. h, i) Transwell migration assays performed in PB or PB-PI3KC2α MDA-MB-468(h) and PB or PB-PI3KC2α 4T1(i). Scale bar = 100 μm. j) Transwell migration and invasion assay performed in MDA-MB-231 treated with control siRNA or siRNA targeting PI3KC2α. Scale bar = 100 μm. All results are shown as mean of at least three independent experiments ± SEM (n.s., no significance, $^{*}P<0.05$; $^{**}P<0.01$).

Figure S3

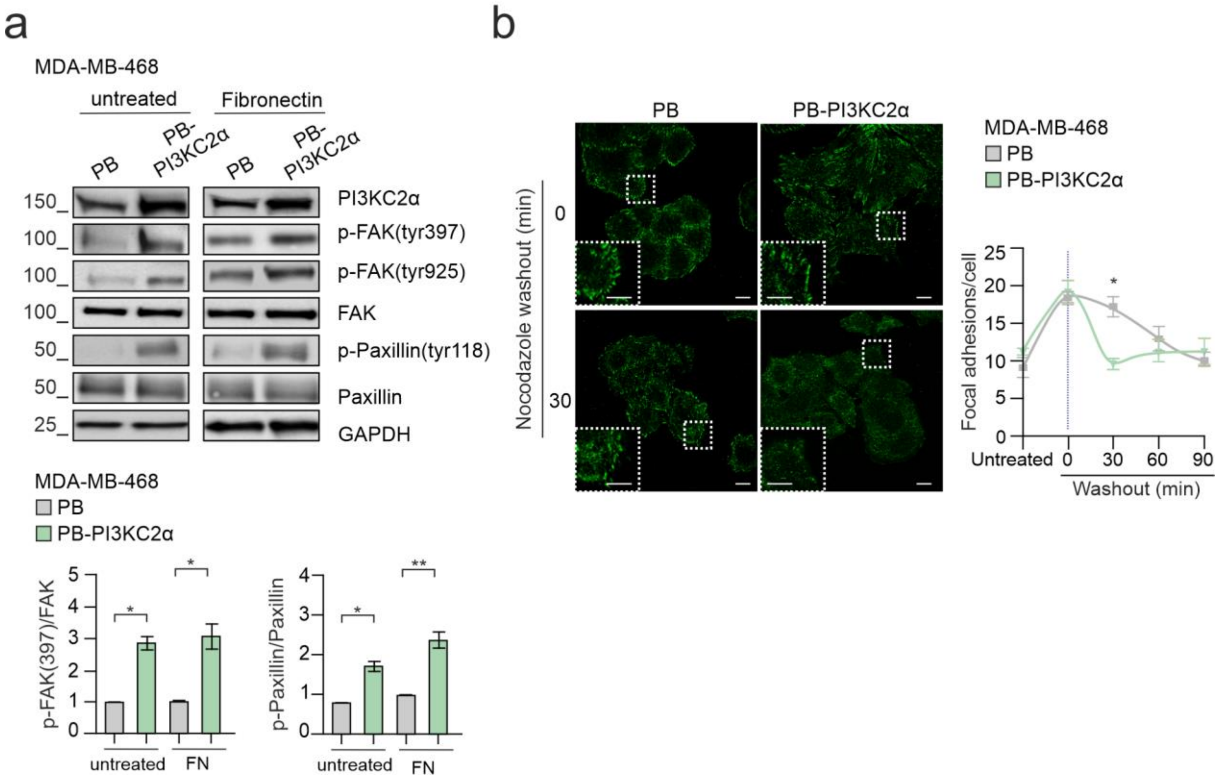

Figure S3. a) Immunoblot analysis (upper panels) and p-FAK(tyr397) and p-Paxillin(tyr118) protein quantification (lower panels) performed on untreated or fibronectin treated PB and PB-PI3KC2α MDA-MB-468. b) Nocodazole washout assay was used to assess focal adhesion disassembly time (see Experimental Section). PB and PB-PI3KC2α MDA-MB-468 were fixed at indicated time points and immunostained for Vinculin. Representative pictures (left) and quantification of focal adhesions number per cell are shown (right). At least 6 images per time point were analyzed (5–10 cells per picture). Scale bar, 10 μm. All results are shown as mean of at least three independent experiments ± SEM (n.s., no significance, *P<0.05; **P<0.01).

Figure S4

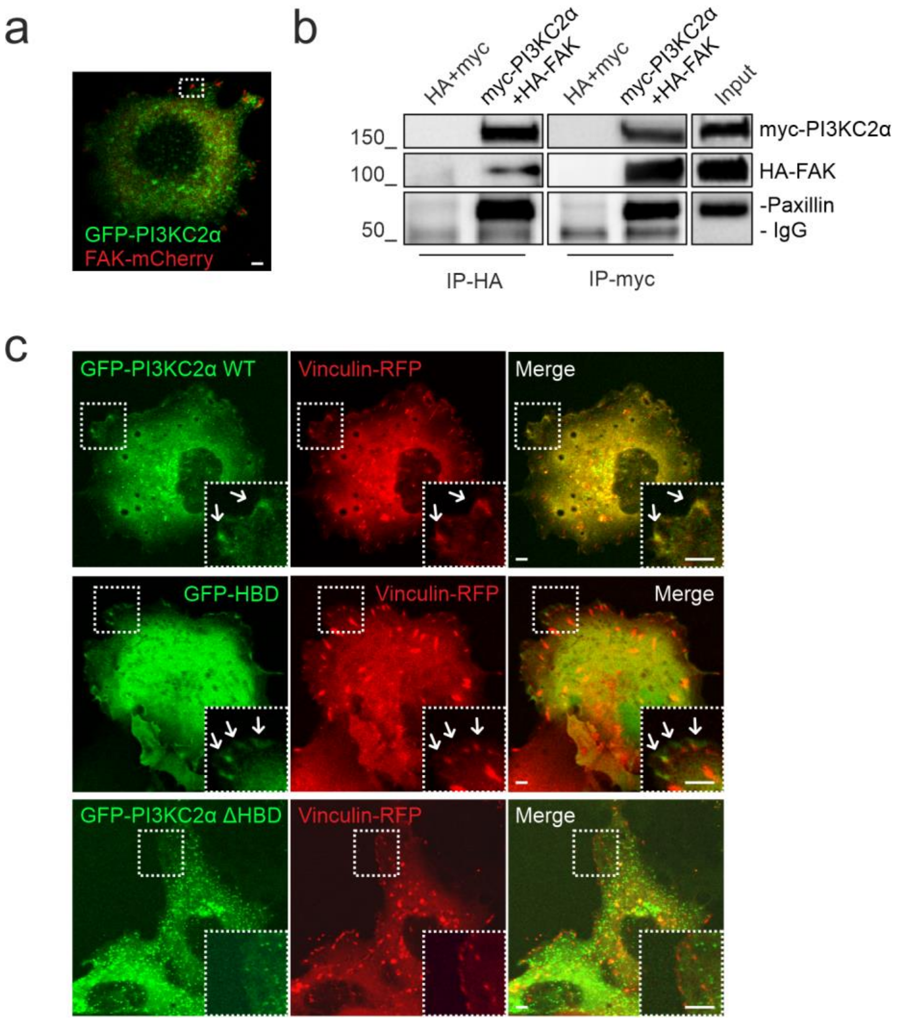

Figure S4. a) Representative picture of live cell imaging performed on MCF7 expressing FAK-mCherry (red) and GFP-PI3KC2α (green). b) Co-immunoprecipitation (Co-IP) experiment performed on HEK293T transfected with HA-FAK and myc-PI3KC2α. Bound proteins were blotted with anti-myc, anti HA and anti-paxillin antibodies. c) Live-cell imaging performed in COS7 expressing Vinculin-RFP (red) and GFP-PI3KC2a (WT, HBD or HBD deleted mutant).

Figure S5

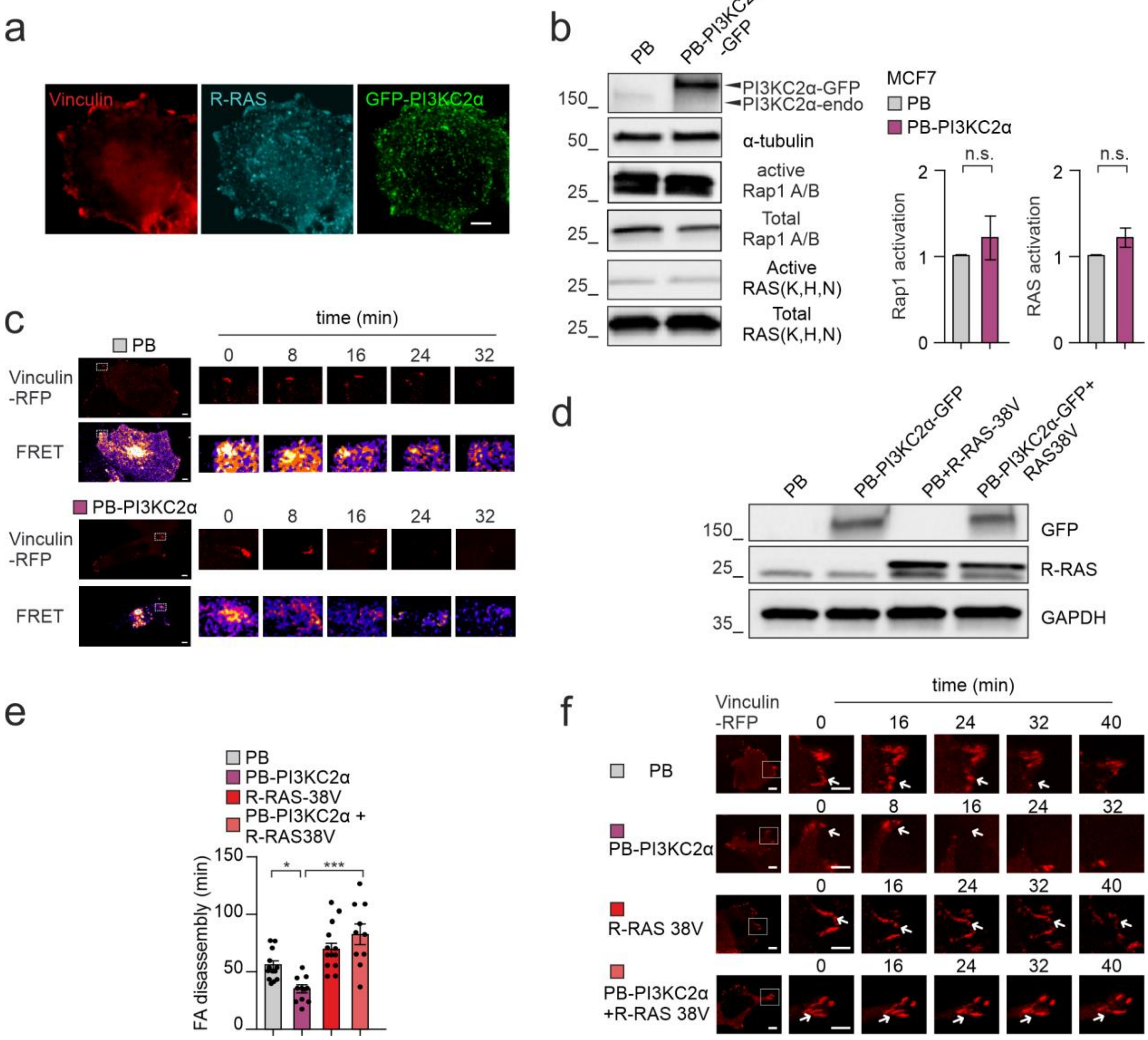

Figure S5. a) Immunofluorescence staining performed on MCF7 showing colocalization of GFP-PI3KC2α (green) with endogenous R-RAS (cyan) and Vinculin (red). b) Pull-down assay (left) and quantification (right) showing active Rap1A/B and RAS (K, H, N) in PB or PB-PI3KC2α MCF7. c) Representative pictures of R-RAS FRET ratio (pseudo-colour) on focal adhesion (red) during its disassembly performed in PB or PB-PI3KC2α MCF7 expressing Vinculin-RFP and Raichu-R-RAS FRET probe. d) Representative immunoblot showing overexpression of R-RAS-38v in PB or PB-PI3KC2α MCF7. e, f) Quantification (f) of focal adhesion disassembly time from time lapse (e) performed on living MCF7 cells expressing Vinculin-RFP and PB, PB-PI3KC2α, R-RAS 38V or PB-PI3KC2α + R-RAS 38V, respectively. n ≥ 10 focal adhesions from three independent experiments. All results are shown as mean of at least three independent experiments ± SEM (n.s., no significance, *P<0.05; ***P<0.001).

Figure S6

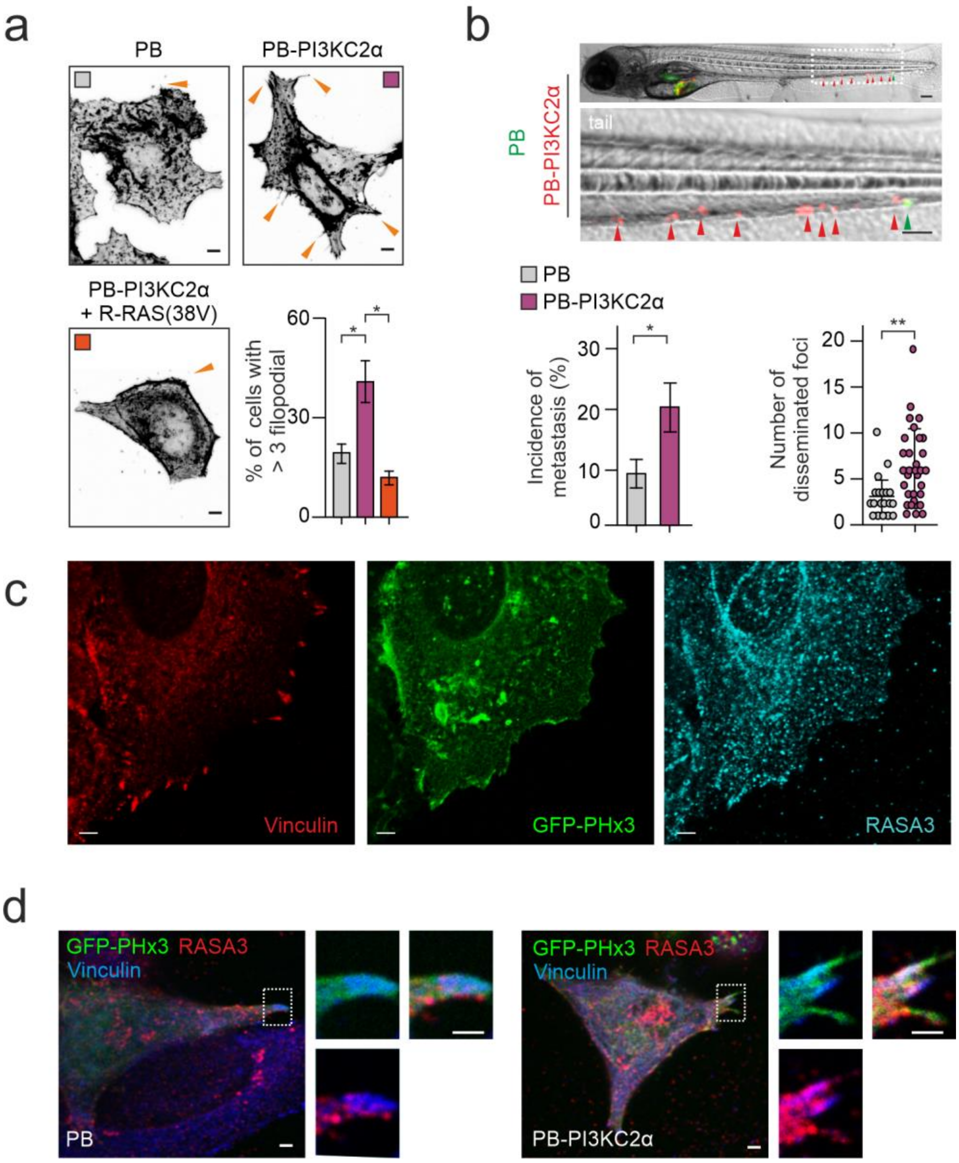

Figure S6. a) Percentage of cells showing filopodia number > 3 were counted in PB, PB-PI3KC2α and PB-PI3KC2α+R-RAS38V MCF7. n≥20 cells from at least three independent experiments. b) Representative pictures (upper panels) and quantification (bottom panels) showing number of disseminated foci and incidence of metastases in zebrafish injected with PB (Green) or PB-PI3KC2α (red) MCF7. Arrows indicate metastatic foci in zebrafish tail. n = 19 (PB), n = 32 (PB-PI3KC2α) zebrafish. Scale bar = 200 µm or 25 µm in enlarged sections. c) Immunofluorescence staining performed on MCF7 showing colocalization of GFP-TAPP1-PHx3 (green) with endogenous RASA3 (cyan) and FAK (red). Scale bar = 10 µm. d) Representative picture of RASA3 (red) at focal adhesion (vinculin, blue) performed on PB or PB-PI3KC2α MCF7 expressing GFP-TAPP1-PHx3 (green). Scale bar, 10 µm.

All results are shown as mean of at least three independent experiments ± SEM (n.s., no significance, *P<0.05; **P<0.01).

Table S1. Primers and probes used in quantitative RT-PCR.

**Gene**	**Left primer(5'-3')**	**Right primer(5'-3')**	**Probe**
CDH1	cccgggacaacgtttattac	gctggctcaagtcaaagtcc	#35
MMP1	gctaacctttgatgctataactacga	tttgtgcgcatgtagaatctg	#7
MMP9	gaaccaatctcaccgacagg	gccacccgagtgtaaccata	#6
Snai1	tacagcgagctgcaggact	atctccggaggtgggatg	#11
Snai2	tggttgcttcaaggacacat	gcaaatgctctgttgcagtg	#7
ZEB1	tgactatcaaaaggaagtcaatgg	gtgcaggagggacctcttta	#31
